# Cost-effectiveness analysis of the treatment of posttraumatic stress disorder related to childhood abuse: comparison of phase-based treatment and direct trauma-focused treatment

**DOI:** 10.3389/fpsyg.2024.1310372

**Published:** 2024-06-21

**Authors:** Noortje I. van Vliet, A. Dennis Stant, Rafaele Huntjens, Maarten K. van Dijk, Ad de Jongh

**Affiliations:** ^1^Dimence Mental Health Group, Deventer, Netherlands; ^2^Zovon, Enschede, Netherlands; ^3^Department of Experimental Psychotherapy and Psychopathology, University of Groningen, Groningen, Netherlands; ^4^Department of Social Dentistry and Behavioral Sciences, University of Amsterdam and Vrije Universiteit, Amsterdam, Netherlands; ^5^School of Health Sciences, University of Salford, Manchester, United Kingdom; ^6^Institute of Health and Society, University of Worcester, Worcester, United Kingdom

**Keywords:** PTSD, CEA, childhood abuse, EMDR, STAIR

## Abstract

**Background:**

Policymakers, health insurers, and health care providers are becoming increasingly interested in cost-effectiveness analyses (CEA’s) when choosing between possible treatment alternatives, as costs for mental health care have been increasing in recent years.

**Objective:**

The current study compared the cost-effectiveness and cost-utility of a phased-based treatment approach that included a preparatory stabilization phase with direct trauma-focused treatment in patients with PTSD and a history of childhood abuse.

**Methods:**

A cost-effectiveness analysis was conducted based on data from a randomized controlled trial of 121 patients with PTSD due to childhood abuse. A phase-based treatment (Eye Movement Desensitization and Reprocessing [EMDR] therapy preceded by Skills Training in Affect and Interpersonal Regulation [STAIR]; *n* = 57) was compared with a direct trauma-focused treatment (EMDR therapy only; *n* = 64). The primary outcome of cost-effectiveness was the proportion of patients with remitted PTSD. Quality-adjusted life years (QALY) were used as the primary outcome measure for cost-utility analysis.

**Results:**

Although the results of the cost-effectiveness analyses yielded no statistically significant differences between the two groups, the mean societal costs per patient differed significantly between the STAIR-EMDR and EMDR therapy groups (€19.599 vs. €13.501; M cost differences = €6.098, CI (95%) = [€117; €12.644]).

**Conclusion:**

STAIR-EMDR is not cost-effective compared with EMDR-only therapy. Since trauma-focused treatment is less time-consuming, non-trauma-focused phase-based, treatment does not seem to be a viable alternative for the treatment of PTSD due to adverse childhood events.

**Clinical trial registration**: https://onderzoekmetmensen.nl/nl/trial/22074, identifier NL5836.

## Highlights

- One of the first studies to compare the cost-effectiveness and cost-utility of phase-based treatment (STAIR-EMDR) with direct trauma-focused treatment (EMDR) in patients with PTSD due to a history of childhood abuse.
- STAIR-EMDR was not cost-effective compared to EMDR only therapy.

## 1 Introduction

Post-traumatic stress disorder (PTSD) is a mental health condition that may result from one or more traumatic events and is characterized by intrusive and recurrent memories of trauma, avoidance of trauma-related stimuli, numbing and/or negative changes in mood or cognition, and changes in reactivity and arousal (American Psychiatric Association, 2017). PTSD has been found to have a major impact on work disability and quality of life (Von der Warth et al., 2020), and may therefore lead to functional impairment and reduced societal productivity (Alonso et al., 2004), resulting in economic burden (Von der Warth et al., 2020). This can result in functional impairment and reduced societal productivity (Alonso et al., 2004). However, frequent physical and mental comorbidities also exert a strong socioeconomic influence on individuals with PTSD (Pacella et al., 2013). Owing to the impact of PTSD on societal costs and quality of life, the APA PTSD Treatment Guidelines (American Psychological Association, 2013) and WHO Guidelines for the Management of Conditions Specifically Related to Stress (World Health Organization, 2013) emphasize the importance of cost-effectiveness studies for future treatment guideline recommendations.

Only one study has systematically reviewed economic evaluations and cost analyses, using PTSD as a diagnostic criterion (Von der Warth et al., 2020). Of the 31 included studies, only 13 performed a full economic evaluation with cost-effectiveness and cost-utility analyses, indicating that intervention costs were measured in relation to effectiveness. Only four of these studies were performed in the European healthcare system. Of the 13 studies with a fully performed economic evaluation, only two also calculated costs from a societal perspective, as recommended by international guidelines (Le et al., 2014; Chang et al., 2018), instead of only a mental health payer’s perspective. In addition, some evidence-based PTSD treatments, including EMDR therapy, were not included in the review (Von der Warth et al., 2020), whereas EMDR therapy was found to be the most cost-effective PTSD treatment among the 10 different PTSD treatments in another large study (Mavranezouli et al., 2020). Thus, health economic evaluations of PTSD therapies from a societal perspective are lacking in Europe. The latter may even be more true for severe forms of PTSD, for which patients with a history of childhood abuse are at risk (Cloitre et al., 2012; Rink and Lipinska, 2020).

There is an ongoing debate about the treatment of individuals with PTSD due to childhood abuse, which revolves around the question of whether they need phase-based treatment instead of treatment according to international treatment guidelines for PTSD. The recommended evidence-based trauma-focused treatments include EMDR and prolonged exposure therapy, which directly target traumatic memories (Cloitre, 2015; De Jongh et al., 2016). According to the ISTSS expert consensus guidelines published in 2012 (Cloitre et al., 2012) the main focus of Phase 1 of a phase-based treatment protocol should be to ensure patients’ safety and teach them emotional and social competencies. The focus of Phase 2 is the processing of traumatic memories whereas that of Phase 3 involves a consolidation of the treatment gains. In response to critical analyses of the ISTSS expert consensus guidelines released in 2012 (De Jongh et al., 2016) a more recent guideline position paper (International Society for Traumatic Stress Studies, 2018), emphasized the importance of personalized treatment by tailoring interventions to the individual needs, instead of using a strict (sequential) treatment program. Nevertheless, it is still unclear whether the addition of a preparation phase (Phase 1) before the trauma-focused treatment is cost-effective compared with direct trauma-focused treatment. Skills Training in Affect and Interpersonal Regulation (STAIR) is the most extensively studied protocol to use as Phase 1 (Cloitre et al., 2002, 2010; Haasija and Cloitre, 2015). The therapeutic objectives of STAIR include: (1) promoting emotional awareness of feelings and their triggers in daily life, (2) teaching emotion regulation strategies, (3) encouraging the adaptive utilization of emotions and enhancing distress tolerance, (4) supporting the identification and modification of dysfunctional interpersonal schemas, (5) facilitating the identification of adaptive and achievable social goals with in various relationships and interpersonal contexts, and, (6) achieving a sense of self-efficacy in both emotional and social domains (Haasija and Cloitre, 2015).

Opponents of the addition of a preparation phase prior to a trauma-focused treatment argue that just targeting traumatic memories of patients with symptoms may lead to similar results and that the addition of a preparation phase delays symptom reduction and thereby may even cause unnecessary suffering (De Jongh et al., 2016), eventually leading to higher costs than immediate trauma-focused therapy.

A recent study examined the cost-effectiveness of prolonged exposure (PE) therapy preceded by STAIR among patients with PTSD related to childhood abuse (Kullberg et al., 2023). Unfortunately, the researchers did not answer the question of whether the addition of STAIR to trauma-focused treatment leads to economic benefits because of the skills gained during the preparation phase. However, they replaced part of the trauma-focused sessions with STAIR (eight sessions of STAIR followed by eight sessions of prolonged exposure versus 16 sessions of prolonged exposure), although STAIR was intended for use in addition to trauma-focused treatment (Cloitre et al., 2012), like we used it in our study (16 sessions EMDR preceded by eight sessions of STAIR versus 16 sessions EMDR only). Hence, a study comparing the cost-effectiveness of phase-based and direct trauma-focused treatments is warranted.

The purpose of this study was to perform secondary analyses based on data from our randomized controlled trial (Van Vliet et al., 2021) to assess the cost-effectiveness of a phase-based treatment protocol (i.e., EMDR therapy preceded by STAIR: Skills Training in Affective and Interpersonal Regulation) compared with direct trauma-focused treatment (i.e., EMDR only) in patients with severe PTSD due to repeated sexual and/or physical abuse during childhood, with STAIR as an actual addition to EMDR therapy.

## 2 Methods

### 2.1 Design and participants

Our economic evaluation was focused on the balance between costs and health outcomes of phase-based treatment (STAIR-EMDR; *n* = 57) compared to direct trauma-focused treatment (EMDR therapy; *n* = 64) in individuals with PTSD due to childhood abuse (Van Vliet et al., 2021). For this randomized controlled trial patients were recruited from two mental health organizations in the Netherlands (Dimence GGZ and GGZ Oost-Brabant). After patients signed a written informed consent form, and were eligible to participate in the study (*N* = 121), they were randomly assigned to one of the two treatment conditions. The power calculation was based on a repeated-measures ANOVA, with the treatment condition as the between-subjects factor and time as the within-subjects factor (Van Vliet et al., 2018). The inclusion criteria were (a) age between 18 and 65 years, (b) PTSD as measured by the Clinician-Administered PTSD Scale for DSM-5 (CAPS-5; 21), and (c) PTSD related to repeated sexual and/or physical abuse before the age of 18 years by a caregiver or a person in a position of authority. This was indexed using the LEC-5 (Weathers et al., 2013b). The exclusion criteria were: insufficient mastery of the Dutch language, acute suicidality for which direct crisis intervention was needed (as assessed by item 9 of the Beck Depression Inventory-II; Beck et al., 1996), when patients had received any well-evaluated treatment for PTSD for at least eight sessions in the past year, when they reported being a victim of ongoing physical and/or sexual abuse, in case of severe use of alcohol or drugs, or in case of an intellectual disability. The study design was registered at^1^ NL5836 and approved by the medical ethics committee Twente NL 56641.044.16 CCMO. Details regarding the comparison of the effects of the two conditions have been published previously (Van Vliet et al., 2021).

### 2.2 Interventions

The phase-based intervention involved eight sessions of STAIR and 16 sessions of EMDR therapy, whereas the direct trauma-focused treatment involved only 16 sessions of EMDR therapy. It is worth noting that both interventions were delivered twice a week for 90 min each. Prior to starting treatment, each patient in both treatment arms received a first session of 90 min consisting of psycho-education and determining relevant traumatic experiences to target during the PTSD treatment. STAIR was performed according to the protocol described by Cloitre et al. (2002). EMDR therapy was performed according to the standard EMDR protocol (Shapiro, 2018; De Jongh and Ten Broeke, 2019), which included all eight phases (Shapiro, 2018) with the only exception that the patients did not receive any relaxation or emotion regulation skills training prior to the processing of their memories (for the rationale see De Jongh et al., 2016). To address patients’ anticipatory fear and avoidance behavior, the flash-forward protocol (Logie and De Jongh, 2014) was applied to target patients’ most scary fantasies about what could happen once starting the EMDR therapy (e.g., losing control, getting overwhelmed by disturbing memories, getting raped by the therapist, or psychotic decompensation). During processing, standard cognitive interweaves to open blocked processing were applied as described by the originator (Shapiro, 2018). After treatment, the patients were not allowed to receive psychological therapy during the months follow-up. For a complete description of these two treatments, see Van Vliet et al. (2018).

### 2.3 Outcome measures

Two economic analyses were performed; a cost-effectiveness analysis and a cost-utility analysis. The primary outcome measure of cost-effectiveness analysis was the proportion of participants with remitted PTSD. The presence of PTSD was measured using the CAPS-5 (Weathers et al., 2013a). This interview includes 20 items on a 5-point Likert scale, resulting in a total score between 0 and 80. The CAPS-5 has good psychometric properties (Weathers et al., 2017).

Quality-adjusted life years (QALY) were used as the primary outcome measure for cost-utility analysis. A QALY of 1 assumes a year of life lived in perfect health (1 Year of Life × 1 Utility = 1 QALY) and a score between 0 and 1 indicates a year of life lived in a state of less than this perfect health (Drummond et al., 1997). The economic evaluation was conducted from a societal perspective; relevant costs in and outside the healthcare sector were prospectively assessed for 9 months for all included participants. Costs and health outcomes were not discounted due to a follow-up period of less than 1 year, which is in accordance with Dutch guidelines that advise the adjustment of calculated effects and costs from 1 year to the next to consider any changes (Zorginstituut Nederland, 2015b). QALYs were derived from EQ-5D-3L (EuroQol Group, 1990), which is a commonly applied self-administered instrument. The EQ-5D consists of five dimensions; mobility, self-care, usual activities, pain/discomfort, and anxiety/depression, each with three levels (from no problems to many problems concerning the dimension). Subsequently, utilities were calculated using Dolan’s algorithm (Dolan, 1997). It also includes a VAS that asks participants to rate their health from 0 (worst imaginable health) to 100 (best imaginable health).

### 2.4 Cost study

Supplementary Table S1 provides an overview of the various types of costs assessed during the study (including the follow-up time). Cost aspects directly related to STAIR and EMDR therapy were assessed in detail, including the cost of contact between participants and therapists (individual sessions), supervision of therapists during the study, materials, and housing. The various types of costs within the healthcare sector were related to the range of healthcare service participants used during the study. In addition, various types of costs outside the healthcare sector were assessed. The costs of informal care were based on the monetary valuation of the time invested by relatives or acquaintances in helping or assisting participants (such as household work or visiting healthcare professionals). By means of the friction cost method, the costs of productivity losses due to illness-related absence from work were estimated (Koopmanschap et al., 1995). Furthermore, the costs related to changes in the amount of voluntary (unpaid) work conducted by the participants were assessed, as asked in the commonly applied self-administered instrument.

Information on healthcare consumption was collected using a detailed case record form adapted to the context of the current study. The case record form assessed, among others, admissions to hospitals, contacts with healthcare professionals, and absence from work. The case record form was administered to all participants at baseline, at the end of treatment (2 or 3 months after baseline in the EMDR and STAIR-EMDR groups, respectively), and 6 and 9 months after baseline.

Unit prices (i.e., the price of one unit of each included cost type) were based mainly on Dutch standard prices (Zorginstituut Nederland, 2015a) to facilitate comparisons with other economic evaluations. The true costs of the resources used were estimated when standard prices were not available. All unit prices were based on the price level of the Euro in the year 2020. The reference prices established for previous years were adjusted to the 2020 prices by applying the consumer price index.

The presence of PTSD diagnosis, the EQ-5D-3L and the case record form for health care consumption were administered to all participants at baseline, at the end of treatment (2 or 3 months after baseline in the EMDR therapy and STAIR-EMDR groups respectively), and 6 and 9 months after baseline.

### 2.5 Economic analyses

The economic evaluation design included cost-effectiveness and cost-utility analyses. In these types of analyses, costs and health outcomes are used to calculate the incremental cost-effectiveness ratio (ICER) relative to one or more alternatives (Drummond et al., 2005). The formula used for calculating the ICER is presented below (with the proportion of participants with remitted PTSD as the outcome measure).


$$\mathrm{ICER}=\frac{\left(C_{\mathrm{STAIR}-\mathrm{EMDR}}–C_{\mathrm{EMDR}}\right)}{\left({\mathrm{PTSD}}_{\mathrm{STAIR}-\mathrm{EMDR}}–{\mathrm{PTSD}}_{\mathrm{EMDR}}\right)}$$

C_STAIR-EMDR_ = mean costs in the STAIR-EMDR group

C_EMDR_ = mean costs in the EMDR group

PTSD_STAIR-EMDR_ = proportion of participants with remitted PTSD in the STAIR-EMDR group

PTSD _EMDR_ = proportion of participants with remitted PTSD in the EMDR group

### 2.6 Statistical procedures

The bootstrap method (Efron and Tibshirani, 1993) was applied to provide information on the uncertainty of the results of the economic evaluation. To deal with participants for whom not all data were available for various measurements, multiple imputation with a bootstrap approach (Oostenbrink and Ai, 2005) was used. In the planned sensitivity analysis, an alternative approach for handling missing data was applied to verify the results.

ICERs were calculated for each of the 2,500 bootstrap iterations and simulated values of the mean estimates for the cost and outcome differences were added to the cost-effectiveness planes (Black, 1990). Finally, cost-effectiveness acceptability curves (CEACs; Fenwick et al., 2004) were calculated. CEACs inform decision makers on the probability that an intervention will be cost-effective, which depends on the willingness to pay per additional unit of health outcome.

Confidence intervals for cost and effect differences were assessed using bootstrap techniques. Cost outcomes in the EMDR group assessed for 2 months between the baseline and the end of treatment were extrapolated to 3 months. The analyses were conducted using SPSS (version 25), R (2022), and CEA-plus (version 2.1).

## 3 Results

Of the 121 participating patients, 40 completed the entire treatment (15 in the STAIR-EMDR condition and 25 in the EMDR condition), 58 lost their PTSD diagnosis before the end of the maximum number of treatment sessions (i.e., early completers; 29 in the STAIR-EMDR condition and 29 in the EMDR-only condition), and 23 dropped out of treatment before the maximum number of sessions were reached, without losing their PTSD diagnostic status (13 in the STAIR-EMDR condition and 10 in the EMDR-only condition). In the STAIR-EMDR condition, one serious non-study-related adverse event was reported, which included a short hospitalization after a suicide attempt. In the EMDR condition two non-study-related adverse events were reported (one due to increased suicidal ideations during the follow-up, and one due to increased psychotic experiences after changes in medication).

### 3.1 Costs and healthcare utilization

A selection of the various types of costs (in and outside the healthcare sector) generated by the two groups during the 9 months of the study is presented in Supplementary Table S2. Only the most relevant cost types, or those that contributed considerably to the total costs (≥ 1% of the total costs in at least one group), are presented here. These costs are based on the data of participants for whom at least one cost measurement was available during the study (for participants who did not use specific types of costs or information was missing, and €0 was applied when calculating group means for this overview).

Supplementary Table S2 also displays information on the utilization of healthcare services; the percentage of participants using each cost type is provided. The mean costs directly related to the studied interventions were €2.436 and € 1.686 per participant in the STAIR-EMDR and EMDR groups, respectively. Costs related to hospital admissions, sheltered living, psychologist contacts, and psychotherapist contacts contributed considerably to the overall costs within the healthcare sector. Outside the healthcare sector, costs related to informal care and productivity losses were relatively high.

### 3.2 Total costs

An overview of the mean total societal costs during the various measurement periods of this study is provided in Table 1. In addition, the number of participants available for each measurement is presented.

**Table 1:** Mean total societal costs (€) during the study.

Measurement (in months)	STAIR-EMDR		EMDR		Mean cost differences (95% CI)^2^
	Mean total costs	*n*	Mean total costs	*n*	
0–3	€12,682	33	€8,261	44	€4,421 (€491, €4,609)
3–6	€4,493	35	€3,126	41	€1,367 (−€562, €2,170)
6–9	€2,734	41	€1,755	38	€979 (−€206, €1,251)
0–9^1^	€19,599	38	€13,501	41	€6,098 (€117, €12,644)

^1^Mean total societal costs during 9 months, estimates based on the multiple imputation plus bootstrap approach used to account for missing data. ^2^95% confidence interval (CI) for the mean cost differences between the groups, Lower and upper boundaries of the CI are presented.

The mean total societal costs of the STAIR-EMDR group were significantly higher than those of the EMDR group for the 0–3 months measurement (as demonstrated by the 95% CI). The differences in mean total societal costs between the groups were not statistically significant for the two subsequent measurements. The mean total societal costs during the 9 months of the study were 19.599 and 13.501 for the STAIR-EMDR and EMDR therapy groups, respectively. The difference between the groups in mean total societal costs during the 9 months was statistically significant.

### 3.3 Health outcomes

The results of the health outcomes of the participants included in the economic analyses are presented in Table 2.

**Table 2:** PTSD outcomes and QALYS during 9 months^1^.

Outcome measure	STAIR-EMDR		EMDR		Mean differences (95% CI^2^)
	Mean (SD)	*n*	Mean (SD)	*n*	
PTSD^3^	0.83 (0.06)	38	0.64 (0.08)	41	0.19 (−0.01, 0.39)
QALY	0.43 (0.04)	30	0.45 (0.03)	35	−0.02 (−0.13, 0.09)

^1^ Estimates were based on the multiple imputation plus bootstrap approach used to account for missing data. ^2^ 95% confidence interval (CI) for the mean difference between groups. Lower and upper boundaries of the CI are presented. ^3^ Proportion of participants with remitted PTSD (at the last measurement).

Analyses of the included outcome measures, remitted PTSD, and QALYs revealed no statistically significant differences between the two groups. PTSD outcomes tended to favor the STAIR-EMDR group; however, QALY outcomes were slightly worse in this group.

### 3.4 Cost-effectiveness analyses

**Figure 1:**
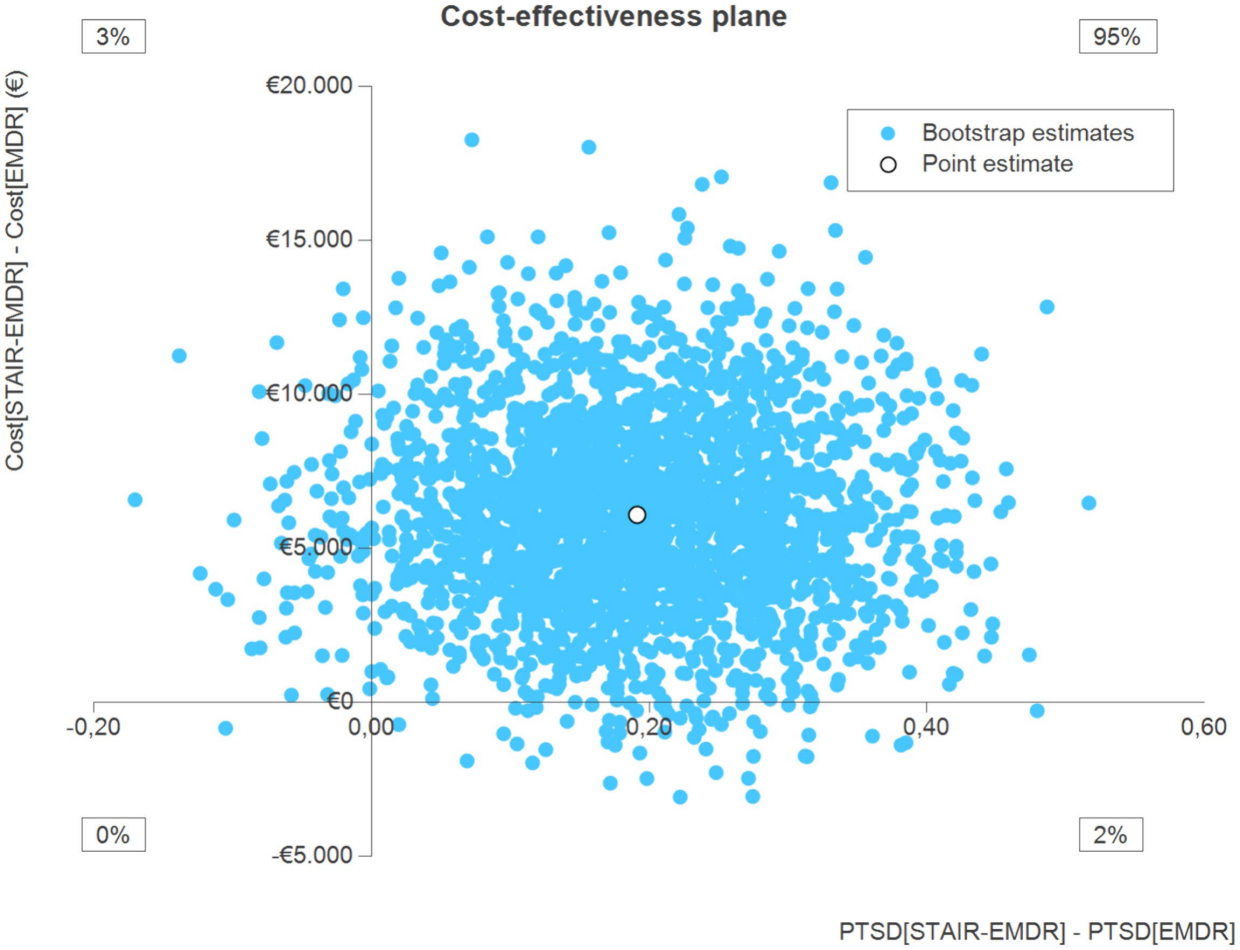
Cost-effectiveness analysis with remitted PTSD as outcome measure.

The cost-effectiveness analyses were based on the data of participants for whom sufficient information was available on both costs and health outcomes (at least 50% of the data available). The results of the cost-effectiveness analysis with remitted PTSD as the primary outcome measure are presented in the cost-effectiveness plane (CEP) in Figure 1. Information is provided on the point estimate of the ICER, and percentage of bootstrap simulations located in each quadrant of the CEP.

**Figure 2:**
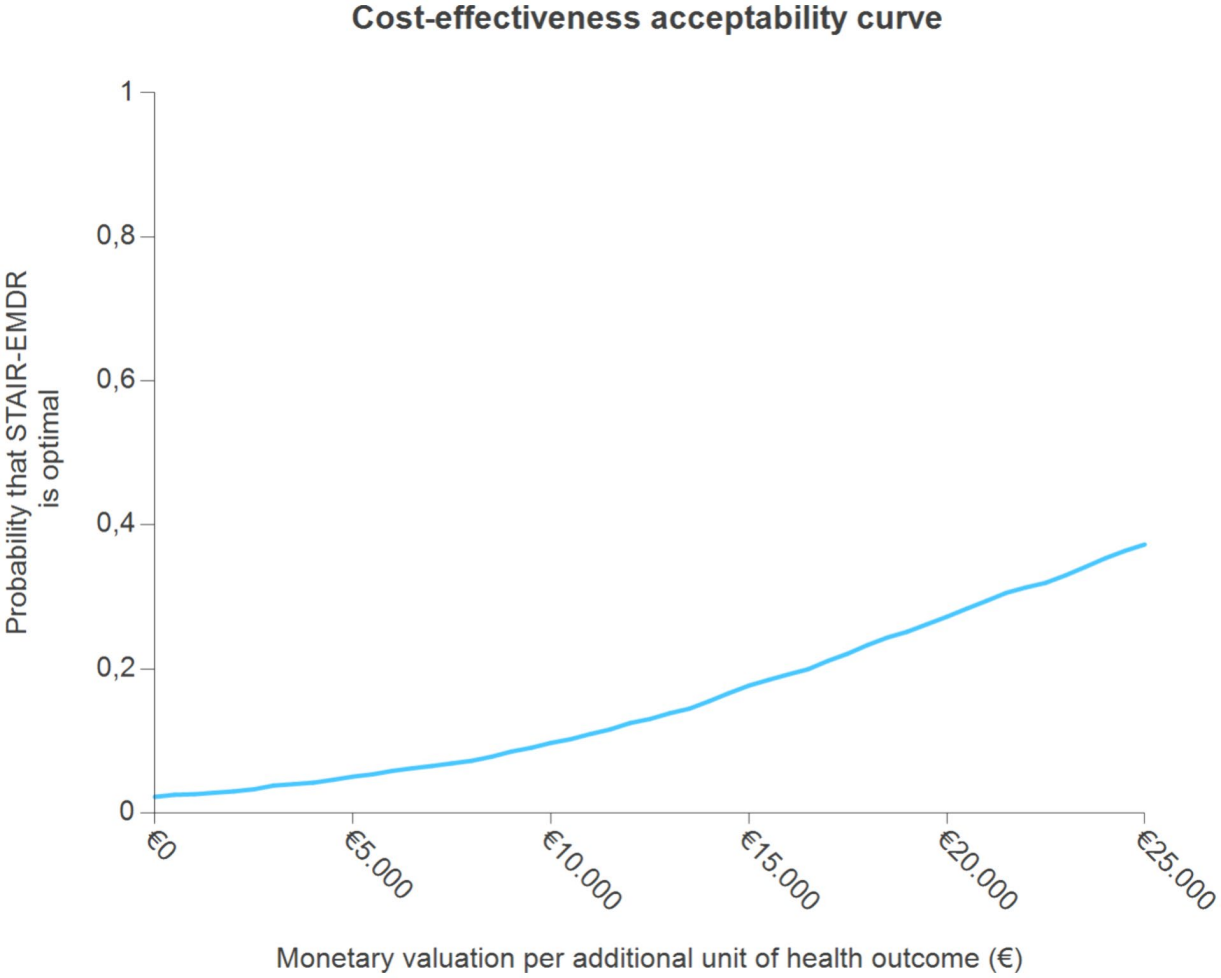
Cost-effectiveness acceptability curve (with remitted PTSD as primary outcome).

The point estimate is located in the northeast quadrant, which indicates higher costs and better PTSD outcomes in the STAIR-EMDR group. Approximately 95% of bootstrap simulations were located in the northeast quadrant. Interpretation of the results of this cost-effectiveness analysis depends on how much decision makers are willing to pay for an additional unit of health outcome (remitted PTSD). The probability that the intervention will be optimal for increasing willingness to pay per additional unit of health outcome indicates that STAIR-EMDR is not likely to be cost-effective compared to EMDR (Figure 2). The probability that STAIR-EMDR is optimal starts at only 0.03 for a monetary threshold of €0, and slowly increases for values up to €25.000. Even at these high monetary values, the probability that STAIR-EMDR is optimal increases to only 0.37.

**Figure 3:**
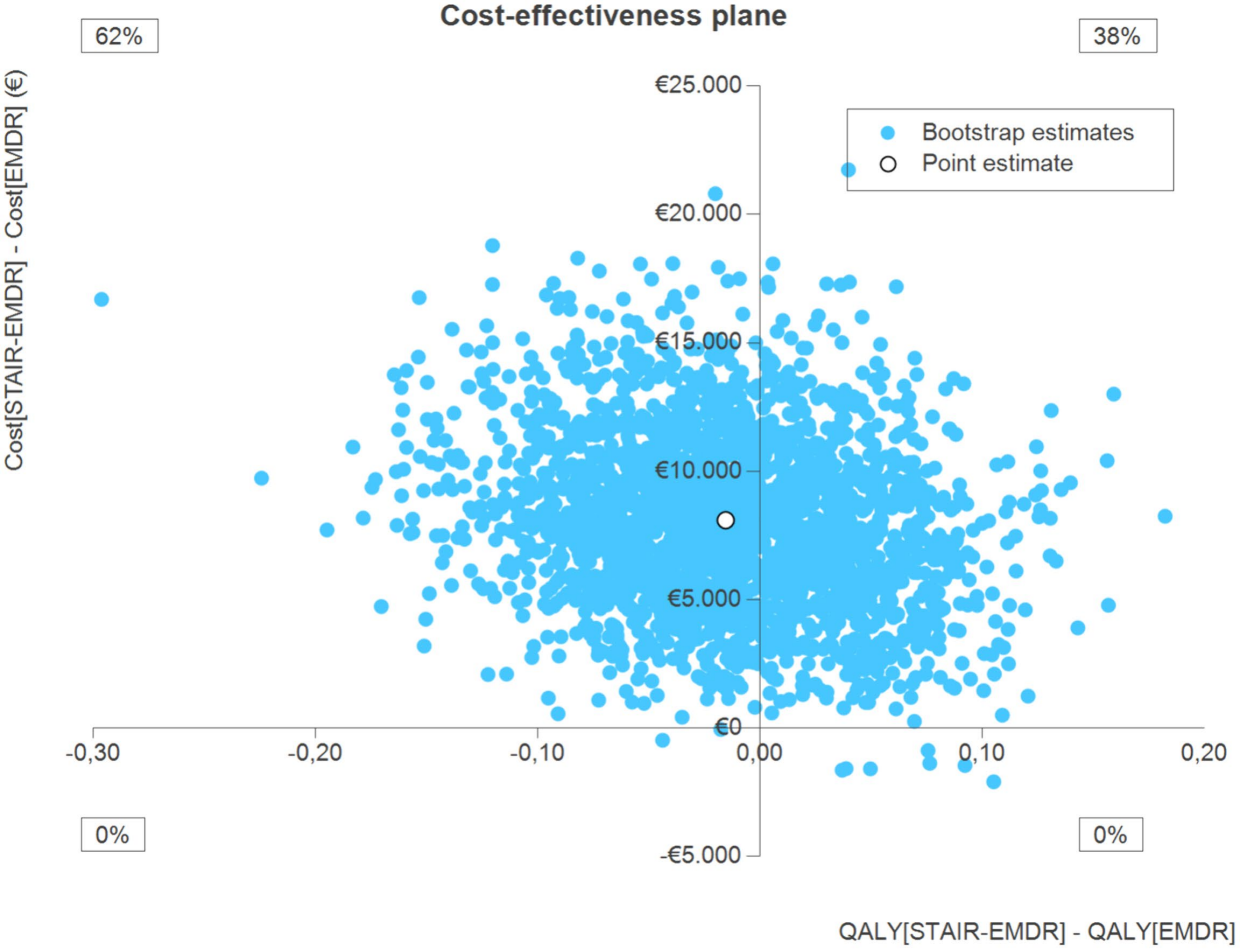
Cost-effectiveness analyses with QALY as outcome measure.

The results of the economic analysis using QALYs as the primary outcome measure are presented in the cost-effectiveness plane in Figure 3. The point estimate is located in the northwest quadrant, which indicates that costs were higher and QALY outcomes were worse in the STAIR-EMDR group. In total 62% of the bootstrap simulations were in the northwest quadrant. Overall, these results indicate that STAIR-EMDR is not cost-effective when focusing on the QALY outcomes.

### 3.5 Sensitivity analysis

In the current study, the data were incomplete for a substantial proportion of the participants. In the planned sensitivity analysis, the influence of the applied approach on dealing with missing data was compared to the results of a complete case analysis. The results indicated that the complete case analysis was associated with a lower probability of STAIR-EMDR being optimal compared to the standard analysis. The results of the sensitivity analysis are therefore not presented in more detail here but are available on request.

## 4 Discussion

To our knowledge, this is the first economic head-to-head comparison of phase-based treatment and direct trauma-focused treatment in patients with PTSD related to childhood abuse, with EMDR as the trauma-focused element and the preparation phase as an actual addition to EMDR therapy. The results indicate that STAIR-EMDR was not cost-effective compared to EMDR therapy alone. The outcome measures of remitted PTSD and QALY’s did not differ significantly between the two treatment conditions, whereas the mean societal costs per patient differed significantly between the STAIR-EMDR and EMDR therapy groups (€19.599 vs. €13.501). The higher societal costs of STAIR-EMDR therapy may be explained by the treatment duration. However, we hoped that these costs caused by treatment duration would have been compensated for by better societal functioning after treatment with STAIR-EMDR, but this was not the case.

In contrast to the purported assumption that STAIR would increase day-to-day functioning by addressing interpersonal and emotion regulation problems (Haasija and Cloitre, 2015), the present results do not show the advantage of adding this treatment to EMDR therapy in terms of cost-effectiveness. In contrast to the study by Kullberg et al. (2023), we found a significant difference in mean societal costs between phase-based and direct trauma-focused conditions, with higher societal costs for phase-based treatment. The costs for PE and STAIR-PE appeared to be much higher (€ 4,479 and € 4,464, respectively) than those for EMDR and STAIR-EMDR (€ 1,686 and € 2,436, respectively). This is consistent with the conclusion of Mavranezouli et al. (2020), who found that EMDR is a less expensive intervention than PE.

We found no difference in treatment effects between the two conditions. These outcomes are comparable to those reported in a previous study (Raabe et al., 2021). The QALY’s from the present study for both STAIR-EMDR and EMDR therapies alone were comparable to the QALY’s for EMDR calculated by Mavranezouli et al. (2020). However, the QALY outcomes in the study by Mavranezouli et al. were measured over a different period of time (3 years) than in our study (9 months), so in comparing both outcomes, many assumptions had to be made, which leaves much uncertainty. The QALY’s gained with EMDR seem somewhat lower than those for Prolonged Exposure from the study by Kullberg et al. (2023); however, also in this case, many assumptions had to be made.

Both operationalizations (loss of diagnosis and increased quality of life) are important intended outcomes, but reducing complaints may be the most import goal, because we can assume that this will lead to an improvement in terms of quality of life, whereas loss of diagnosis (no longer meeting all diagnostic criteria of a mental health condition) will in many cases mean in practice that at least a part of the symptoms persist (Schnurr and Lunney, 2019).

A strength of the present study is that we performed a full economic evaluation with a cost-effectiveness analysis and a cost-utility analysis of both treatment forms, evaluating the costs in relation to the effectiveness of the interventions, as recommended by the national and international guidelines for health cost evaluations (European Network for Health Technology Assessment, 2015; Zorginstituut Nederland, 2015b). Second, in addition to health care costs, societal costs such as productivity losses and care by relatives or acquaintances were assessed. These costs reflect the use of resources from other sectors in society (European Network for Health Technology Assessment, 2015). By considering these costs, we avoided artificially lowering costs by shifting medical costs to informal societal care costs.

One limitation of this study was the number of missing values, which may have limited the power of the statistical calculations, leading to less reliable outcomes. Of the 57 participants in the STAIR-EMDR condition and 64 participants in the EMDR therapy condition, only 38 (66.7%) and 41 (64.1%) participants, respectively, remained in the cost-effectiveness measurements at the 6-month follow-up. Because we used advanced methods to deal with incomplete data, patient data could still be included in our analyses when at least half of the measurements were available. A second limitation is the relatively short study period (9 months), which precludes the visibility of societal gains in the long term. For decision makers, outcomes assessed over longer periods (at least 1–2 years) may prove more relevant for policy decisions.

In conclusion, although phase-based treatment and EMDR therapy alone demonstrated no difference in effectiveness in achieving remission of PTSD symptoms and improving Quality-Adjusted Life Years, the societal costs of phase-based treatment were found to be significantly higher than those of trauma-focused therapy. Where cost and time are issues, EMDR therapy alone is the treatment of choice. However, some individuals might benefit from a longer treatment period with an additional focus on clinically relevant symptoms, such as dissociative sequelae.

## Data availability statement

The raw data supporting the conclusions of this article will be made available by the authors, without undue reservation.

## Ethics statement

The studies involving humans were approved by Medical Ethic Committee Twente (merged with the Institutional Review Board United), reference number P16–03. The studies were conducted in accordance with the local legislation and institutional requirements. Written informed consent for participation in this study was provided by the participants/patients.

## Author contributions

NV: Conceptualization, Data curation, Funding acquisition, Investigation, Methodology, Project administration, Writing – original draft, Writing – review & editing. AS: Funding acquisition, Supervision, Formal analysis, Methodology, Supervision, Writing – review & editing. RH: Conceptualization, Funding acquisition, Supervision, Writing – review & editing. MD: Conceptualization, Funding acquisition, Supervision, Writing – review & editing. AJ: Conceptualization, Funding acquisition, Supervision, Writing – review & editing.

## Footnotes

^1^ https://onderzoekmetmensen.nl/nl/trial/22074
