# Supplementary material for: Cost-effectiveness analysis of the treatment of posttraumatic stress disorder related to childhood abuse: comparison of phase-based treatment and direct trauma-focused treatment
Source: Front Psychol. 2024 Jun 21;15:1310372. doi: 10.3389/fpsyg.2024.1310372 (PMC11224530; doi:10.3389/fpsyg.2024.1310372)
Supplement: Supplementary file 1 [file Data_Sheet_1.docx]

Supplemental Table 1. Types of costs included in the analyses

| Costs within the healthcare sector | Costs outside the healthcare sector |
| --- | --- |
|  |  |
| Interventions (including STAIR and EMDR contacts, supervision, housing) | Informal care |
| Inpatient and semi-inpatient care | Productivity losses |
| Outpatient and community care |  |
| General healthcare |  |
| Medication use |  |

Supplemental Table 2. Costs (€) in and outside the healthcare sector during 9 months

| *Selected cost types* | STAIR-EMDR (n=45) | | EMDR (n=49) | |
| --- | --- | --- | --- | --- |
|  | Mean costs (SD) | %^1^ | Mean costs (SD) | %^1^ |
| *Interventions* |  |  |  |  |
| STAIR-EMDR, EMDR | €2,436 (€874) | 100 | €1,686 (€507) | 100 |
|  |  |  |  |  |
| *(Semi-)inpatient care* |  |  |  |  |
| Hospital admissions | €405 (€1,903) | 11 | €1,187 (€4,625) | 16 |
| Day treatment | €7 (€44) | 2 | €171 (€1,025) | 8 |
| Outpatient clinic | €190 (€384) | 38 | €168 (€297) | 35 |
| Sheltered living | €2,071 (€8,540) | 7 | €142 (€460) | 10 |
|  |  |  |  |  |
| *Outpatient/community care* |  |  |  |  |
| Psychiatrist | €423 (€2,065) | 29 | €204 (€745) | 39 |
| Psychologist | €1,256 (€1,581) | 56 | €2,086 (€1,797) | 73 |
| Psychotherapist | €929 (€1,726) | 27 | €882 (€1,802) | 22 |
| Social worker | €101 (€353) | 18 | €299 (€1,260) | 16 |
| Physiotherapist | €204 (€464) | 38 | €162 (€344) | 29 |
|  |  |  |  |  |
| *Medication use* |  |  |  |  |
| Prescribed medication | €92 (€150) | 64 | €155 (€302) | 65 |
|  |  |  |  |  |
| *Outside healthcare sector* |  |  |  |  |
| Informal care | €2,402 (€3,574) | 58 | €1,849 (€2,871) | 55 |
| Out-of-pocket costs | €150 (€639) | 13 | €188 (€1,271) | 4 |
| Productivity losses paid work | €4,256 (€8,515) | 29 | €1,710 (€4,662) | 20 |

^1^ Percentage of participants using the cost types concerned
